# Cytological and Wet Mount Microscopic Observations Made in Urine of *Schistosoma haematobium*-Infected Children: Hint of the Implication in Bladder Cancer

**DOI:** 10.1155/2019/7912186

**Published:** 2019-09-02

**Authors:** Patience B. Tetteh-Quarcoo, Benjamin K. Akuetteh, Irene A. Owusu, Solomon E. Quayson, Simon K. Attah, Robert Armah, Emmanuel Afutu, Ama Afrah, Kantanka Addo-Osafo, Cecilia Smith, Richard K. Gyasi, Patrick F. Ayeh-Kumi

**Affiliations:** ^1^Department of Medical Microbiology, School of Biomedical and Allied Health Sciences, University of Ghana, Accra, Ghana; ^2^Department of Pathology, School of Biomedical and Allied Health Sciences, University of Ghana, Accra, Ghana

## Abstract

**Background:**

Schistosomiasis is the second major human parasitic disease next to malaria, in terms of socioeconomic and public health consequences, especially in sub-Saharan Africa. *Schistosoma haematobium* (*S. haematobium*) is a trematode and one of the species of *Schistosoma* that cause urogenital schistosomiasis (urinary schistosomiasis). Although the knowledge of this disease has improved over the years, there are still endemic areas, with most of the reported cases in Africa, including Ghana. Not much has been done in Ghana to investigate cytological abnormalities in individuals within endemic communities, although there are epidemiologic evidences linking *S. haematobium* infection with carcinoma of the bladder.

**Aim:**

The aim of this study was to identify microscopic and cytological abnormalities in the urine deposits of *S. haematobium*-infected children.

**Methodology:**

Three hundred and sixty-seven (367) urine samples were collected from school children in Zenu and Weija communities. All the samples were examined microscopically for the presence of *S. haematobium* eggs, after which the infected samples and controls were processed for cytological investigation.

**Results:**

*S. haematobium* ova were present in 66 (18.0%) out of the 367 urine samples. Inflammatory cells (82%, 54/66), hyperkeratosis (47%, 31/66), and squamous cell metaplasia (24%, 16/66) were the main observations made during the cytological examination of the *S. haematobium*-infected urine samples.

**Conclusion:**

Cytological abnormalities in *S. haematobium*-infected children may play an important role in the severity of the disease, leading to the possible development of bladder cancer in later years, if early attention is not given. Therefore, routine cytological screening for urogenital schistosomiasis patients (especially children) at hospitals in *S. haematobium*-endemic locations is recommended.

## 1. Introduction

Schistosomiasis is considered a neglected tropical disease that ranks second after malaria in terms of human suffering, in the tropics and subtropics. The genus *Schistosoma* contains different species that cause acute and chronic infection in humans: *Schistosoma haematobium* (*S. haematobium*), *S. mansoni*, *S. japonicum*, *S. mekongi*, *S. intercalatum*, *S. malayensis*, and *S. guineensis* [1]. *S. haematobium* causes urogenital schistosomiasis (urinary schistosomiasis) [1–3]. In 2016, it was estimated that globally schistosomiasis affects over 200 million people [2]. It is believed that the majority of people living with urinary schistosomiasis are in Africa, including Ghana, and this is evident from numerous prevalence studies conducted across the country [3–7]. Urogenital schistosomiasis has been reported to be higher in children than in adult [8]. Even though there has been an improvement in knowledge of urogenital schistosomiasis, repeated cases with accompanying morbidities and mortalities are still being reported [9, 10].

The infection of *S. haematobium* still leads to some form of abnormalities, regardless of whether it was during the early stage or not, there is mixed infection with other schistosomes or not, and the quantity of eggs of *S. haematobium* present in the tissues [11]. Mortality may occur from granulomatous inflammation complications [12], renal insufficiency [13], possible bladder cancers [14], and ulceration or depletion of the vesicle, as well as the ureteral wall [12]. Emerging evidence shows that immune regulation of inflammatory response, independent of infectious burden, is linked with the risk of some forms of morbidity due to schistosomiasis infection [15]. Therefore, light infection should not be overlooked as a cause for disability; hence, the recommendation that its prevention be integrated as part of the design for population-based schistosomiasis control programmes [15].

Studies in Africa and other parts of the world have linked squamous cell carcinoma of the urinary bladder with *S. haematobium* infection [14, 16–19]. This association is based on the close correlation of bladder cancer incidence with the prevalence of *S. haematobium* infection within different geographic areas [16–20], as well as expression of markers such as cyclooxygenase-2 (COX-2) and inducible nitric oxide synthase (iNOS) in urinary schistosomiasis patients [21].

Cytopathological examination of urine samples has been considered a routine noninvasive diagnostic procedure used to detect cancer of the urinary tract, primarily bladder cancer [22, 23]. This diagnostic procedure is also used during the follow-up procedures of the patients previously treated for bladder cancer, in order to detect recurrence as early as possible [23]. In the high-risk population, the cytopathological examination of urine is remarkably used for the screening of urothelial carcinoma [22].

Despite the link between *S. haematobium* and bladder cancer, only limited data are available on cytopathological findings in schistosomiasis-related tumors. Meanwhile, damage from *S. haematobium* infection has been attributed to parasite eggs deposited at the affected sites [24]. To the best of our knowledge, there seems not to be a study focusing on cytological examination of urine samples from *S. haematobium*-infected children within endemic communities in Ghana. Therefore, the connection between these cytological abnormalities and the severity of the disease in these areas has not been demonstrated. The current study is among the first to present finding from cytological and wet mount microscopic observations made in the urine of *Schistosoma haematobium*-infected children, living in endemic communities in the capital city of Ghana.

## 2. Materials and Methods

### 2.1. Approach, Study Design, Study Sites, and Scope of the Research

A cross-sectional study was conducted in two communities (Zenu and Weija) of the Greater Accra Region, the most urbanized region in Ghana. The geographical coordinates of the Zenu community are 5°42′0″ North, 0°20′0″ West [7] while those of Weija are 5°34′ North, 0°20′ West, with Weija occupying a total land area of about 341.838 square kilometres [25]. The major economic activities of these communities are subsistent farming and fishing, with a few inhabitants being civil servants. The presence of a lake in the Zenu community and a river in the Weija community (serving as a great source of water supply for various domestic activities) might have attracted most of the settlers [8].

Earlier studies [7, 26] have considered these two communities (Zenu and Weija) as urogenital schistosomiasis-endemic areas, and thus, they were selected for the current study, in order to increase the probability of obtaining *S. haematobium*-infected samples.

After obtaining informed consent, fifty-milliliter (50 ml) clean, dry, leak-proof, wide-mouthed containers were given to each child to provide urine, with adequate instructions. Urine sample collection was done between 10:00 am and 12:00 pm for maximum yield [27]. The urine samples were transported on ice immediately to the Parasitology Laboratory of the Medical Microbiology Department, School of Biomedical and Allied Health Sciences, University of Ghana, for analysis.

The scope of this study was to delineate observations (cytological and wet mount microscopic) in the urine of *Schistosoma haematobium*-infected individual, giving a hint of *S. haematobium* implication in bladder cancer. The study focused on children of school-going age within the selected communities of the Greater Accra Region of Ghana.

### 2.2. Urine Chemistry and Wet Mount Examination

The collected urine samples were analyzed for haematuria and proteinuria, alongside other urine chemistry parameters (pH, specific gravity, glucose, ketones, bilirubin, urobilinogen, and leucocytes) using urine chemistry reagent strips (URIT 10V, URIT Medical Electronic Co. Ltd., China). The results were recorded as negative or positive (trace, +, ++, and +++) [28]. Ten milliliters (10 ml) of the urine sample was transferred into 15 ml centrifuge tube and centrifuged at 300 rpm for 5 minutes. The supernatant was decanted until the one milliliter (1 ml) mark, after which fifty microlitres (50 *μ*l) of the sediment was transferred onto a clean glass microscope slide (Biogenix Inc Private Ltd., Uttar Pradesh, India). The presence and intensity of *S. haematobium* eggs were determined microscopically using the ×10 objective of an optical light microscope (Leica Galen III, catalogue E no. 317506, serial no. ZG6JA4). With the eggs intensity count, the number of eggs present per 10 ml of urine sample was categorized as mild (≤50 ova/10 ml of urine) and heavy (≥50 ova/10 ml of urine) [29].

### 2.3. Cytological Examination

With the urine cytology, 5 ml of the infected and control samples were centrifuged at 1500 rpm for 10 minutes after which the supernatant was decanted leaving about 1.5 ml. The volume was resuspended and then cytocentrifuged 3–5 drops per slide in order to prepare monolayer smear of urine deposits directly onto the slide [30]. The smear slides were wet fixed with 95% ethanol for 15 minutes before staining with Papanicolaou (PAP) stains. Abnormalities such as squamous metaplastic cells, inflammatory cells, and hyperkeratotic cells were examined and reported.

### 2.4. Statistical Analysis

Data obtained were stored in Microsoft Excel and analyzed using the Statistical Package for the Social Sciences (IBM® SPSS® version 20.0). Data were summarized by determining frequencies of abnormalities detected in both urine deposit wet mount examination and cytological examination.

### 2.5. Ethical Statement

This work received ethical clearance from the Ethical and Protocol Review Committee of the College of Health Sciences, University of Ghana (Protocol ID no: MS-Et/M.7-P 3.1/2015-2016). The consent of the students, teachers as well as parents and guardians was sought before sampling.

## 3. Results

Among the 367 urine samples used, 66 were infected with *Schistosoma haematobium*, giving a prevalence of 18% for this study. Out of the 66 samples that contained *Schistosoma* eggs according to the microscopy of the wet mount, when subjected to cytological examination, *S. haematobium* eggs were detected in 40 (40/66, 61%) of them (Table 1).

**Figure 1:**
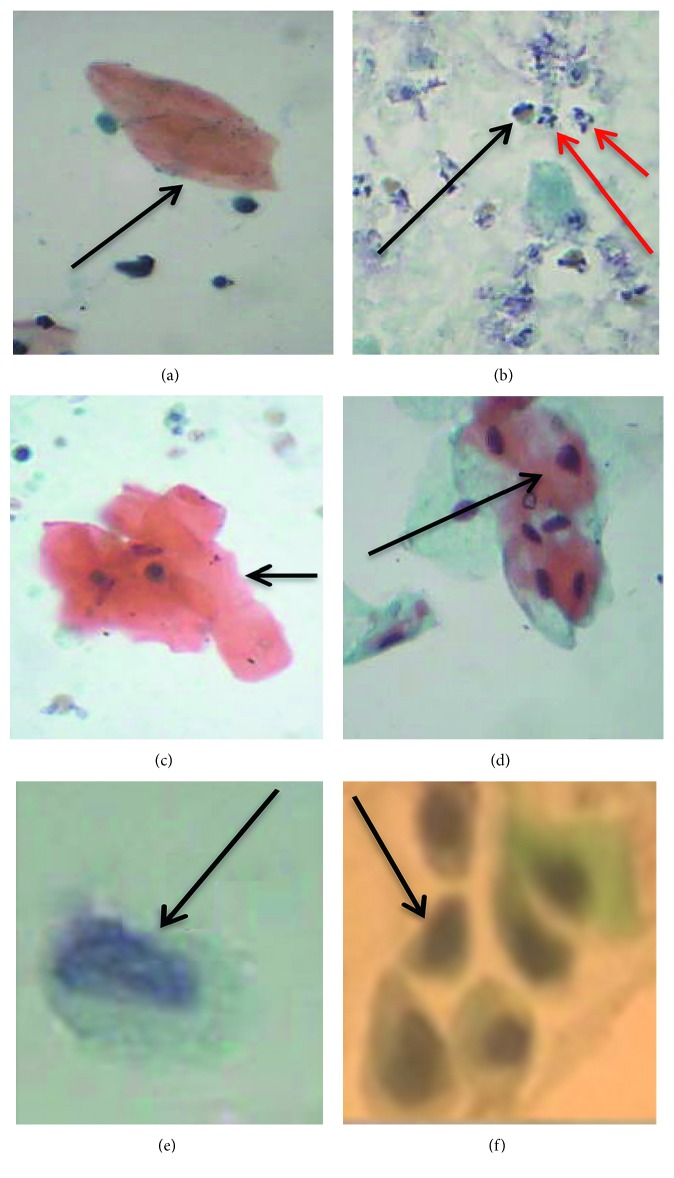
Papanicolaou-stained smears showing cytological observations. (a) Anucleated keratinized cell (arrow—hyperkeratotic cell). (b) Inflammatory cells (red arrows point to neutrophils while black arrow points to eosinophils). (c) Keratinized cell (nucleated). (d) Cluster of keratinized and nonkeratinized cells (arrow pointing to keratinized cells). (e) Reactive urothelial/transitional cells. (f) Squamous metaplastic cells (arrow pointing to one of such cells) (source of images: authors' laboratory work).

Papanicolaou staining of the urine smears revealed different cytological abnormalities (Figure 1). Squamous metaplastic cells (Figure 1(f)), inflammatory cells (Figure 1(b)), and hyperkeratotic cells (Figure 1(a)) were the main cytological observations made in the samples. Urothelial/transitional cells were also found (Figure 1(e)). Inflammatory cells were observed in most (82%) of the urine samples, followed by hyperkeratosis (Table 1). Haematuria was observed by urine reagent strips, wet mount microscopy examination, and cytological examination in 56%, 44%, and 33.3% of the urine samples, respectively (Table 1).

**Figure 2:**
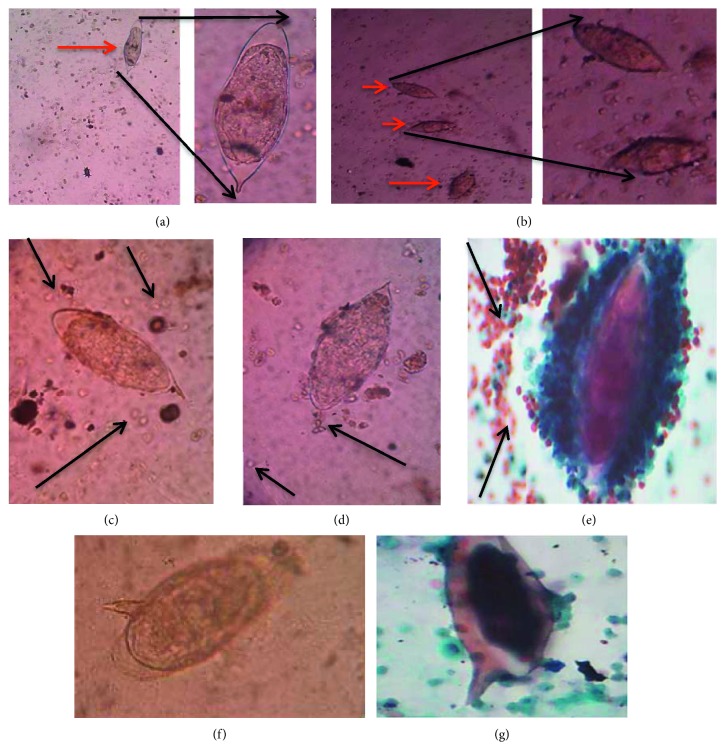
Wet mount and Papanicolaou-stained smears. (a) Low/light intensity of *S. haematobium* eggs (with a zoom in), (b) high intensity of *S. haematobium* eggs in the samples (with a zoom in), (c, d) presence of high and low number of red blood cells observed in wet mount (black arrows pointing to some red blood cells), (e) presence of blood cells in Papanicolaou-stained smears (black arrows pointing to cluster of red blood cells around an *S. haematobium* egg), eggs of *S. mansoni* in the *S. haematobium*-infected urine samples (f) in wet mount and (g) in Papanicolaou-stained smears (source of images: authors' laboratory work).

From the microscopic examination of wet preparation, 47% (31/66) of the samples had high egg intensity (above 50 eggs/10 ml urine), as depicted in Figure 2(b) in comparison with the low intensity represented in Figure 2(a). Both wet mount and cytological examinations showed RBCs in the presence of *Schistosoma haematobium* eggs (Figures 2(c)–2(e)). Out of the sixty-six *S. haematobium*-infected samples, two contained *Schistosoma mansoni* eggs (usually found in stool), in wet mount and cytology stained slides, respectively (Figures 2(f) and 2(g)).

## 4. Discussions

This study found *Schistosoma haematobium* infection in 66 out of the 367 children (representing 18%), indicating that urinary schistosomiasis continues to be a disease of great public health importance, especially in children. *S. haematobium* infection in children has been observed to cause stunting and wasting, which are commonly unacknowledged morbidity [10, 31–33]. In a study by Botelho et al. [32], the proportion of all children and adolescents who had body mass index (BMI) less than 15 kg/m^2^ increased from 57.78% to 66.67% in *S. haematobium*-infected children, who were more stunted and wasted than in noninfected children. The mean weight-for-age Z score (WAZ) among the children in that study was significantly reduced in *S. haematobium*-infected as compared to noninfected [32]. Bustinduy et al. [33] associated wasting with *Schistosoma haematobium* infection in male children even with light intensity of the infection. Our observation therefore supports their suggestion that this vulnerable population urgently needed to be targeted for implementation of measures for treatment and control [33].

The public health importance of this infection was highlighted in this study where the majority of the *S. haematobium*-infected children were found to have the different types of cytological abnormalities such as squamous metaplastic cells which could develop into malignant cells in the continuous presence of a carcinogenic agent such as *S. haematobium*. The International Agency for Research on Cancer classified *S. haematobium* infection as a group 1 biocarcinogen. Schistosoma worm and egg-derived estrogen-like molecules and their metabolites have been postulated as the main carcinogenic substances implicated in schistosomiasis-linked cancers [34]. A retrospective study done in Accra associated 20.5% of cancer cases with tissue schistosomiasis, where the urinary bladder was the most commonly (93.6%) affected organ [31]. Their study also found 98.7% of all tissue schistosomiasis to be caused by *S. haematobium* [31]. With the retrospective study in mind, and the findings from the current study, it can be inferred that the possibility of some of the *S. haematobium*-infected pupils (especially those who had squamous cell metaplasia), having the risk of developing cancer later in life, cannot be overlooked.

While the specimen used for the retrospective study was from clinical cases, seemingly healthy children were used in the current study. Therefore, it is important to note that if this seemingly “benign” *Schistosoma* infection is left untreated in these children now, there is a probability of some of them having a future (with respect to developing cancer) similar to that of the subjects in the study by Der et al. [31].

Ketabchi and Moshtaghi-Kashanian [16] presented a case study involving a 60-year-old man who briefly lived in a schistosomiasis-endemic area of Khuzestan Province (neighbouring province of Persian Gulf in Iran). Approximately 20 years after his short stay at the schistosomiasis-endemic area, he was referred to the Urology Department of Kerman University of Medical Sciences, with haematuria and dysuria. Sonography revealed a polypoid mass on the bladder floor while cystoscopy and biopsy of the bladder tumor showed simultaneous squamous cell carcinoma and transitional bladder cell carcinoma in the presence of *S. haematobium* [35]. They indicated that bladder tumors related to schistosomiasis are mainly squamous cell type and there are rare reports of the simultaneous presence of squamous cell bladder carcinomas and transitional cell carcinoma [35].

Even though this study did not set out to ascertain the mechanism of progression from squamous cell metaplasia to carcinomas, it is important to note that these cellular changes, and abnormalities that precede carcinomas, can be used as markers, hinting of a possible squamous cell bladder carcinoma development early enough and possibly treat or manage before they get to an advanced stage. This will help to avoid the situation in some developing countries, including Ghana, where cancers (including squamous cell bladder carcinomas) seem to be diagnosed at the advanced stage. The use of cytology might therefore be very useful in suspecting early, the development of squamous cell bladder carcinomas associated with *S. haematobium* infection in endemic communities like it was in this study.

Cytopathological examination of urine samples has been described as a routine noninvasive diagnostic procedure used to detect cancer of the urinary tract, primarily, bladder cancer [22, 23]. Thus, it can be applied in the early detection of a developing squamous cell bladder carcinoma, caused by *S. haematobium* infection in endemic communities, as in the current study. Urine cytology has been identified to have good sensitivity (greater than 90%) for high-grade urothelial tumors and carcinoma in situ, among individuals in the United States [36], and has been considered an important adjunct in the evaluation of patients at high risk of urothelial tumors, because of its positive predictive value in such patients [37]. Ninety-seven (97%) specificity for urine cytology have been observed against 63% for ImmunoCyt and 90% for UroVysion in detecting urothelial carcinoma in patients with a previous history of bladder cancer [38]. Also, a positive predictive value of 100% in the diagnosis of urological malignancies has been reported among urinary cytology specimen requests made at the Department of Urology, Royal Sunderland Hospital [39].

The observation that inflammatory cell was the cytological abnormality in the majority of the samples, followed by hyperkeratosis and squamous cell metaplasia, respectively is similar to an earlier study [40]. In that study [40], the prevalence of inflammation was the highest (39%), followed by metaplasia (33%), hyperkeratosis (30%), and frank atypia (0.4%). Meanwhile, in the current study, frank atypia was not observed in any of the samples, but this observation is not surprising, since in the previous study, just a small percentage (0.4%) of frank atypia was observed, in spite of the very high number (*N* = 1,014) of participants used [40].

Even though the link between schistosomiasis and bladder cancer is generally accepted, its carcinogenic mechanisms are less clearly defined [16]. In some cases, severe metaplasia in bladder urothelium may represent a precancerous transformation, while in others it may merely serve as a marker for the prolonged inflammation that is associated with high cancer risk [41–44]. Hyperkeratosis as observed in the current study population has been identified to be linked with cancer formation in patients with chronic irritation due to bladder stones, chronic infection, or prolonged catheterization [45, 46], as well as *S. haematobium* infection [42]. In the capuchin monkey model, Cheever et al. [44] demonstrated that intense *S. haematobium* infection is associated with the development of significant multifocal proliferative lesions that resemble low-grade carcinomas. In these animals, the natural loss of infectious burden after several years was associated with regression of these lesions, indicating that they were noncancerous in nature. However, this sort of proliferative growth, combined with increased excretion and/or local formation of mutagens in the *S. haematobium*-inflamed bladder [45], could be a contributory factor for the onset of cancer formation in humans. The risk of cancer formation is said to be greater when chronic inflammation is combined with exposure to urinary carcinogens [46].

The observation of high number of eggs coupled with blood in urine is not strange, since these have always been associated with the infection and the disease severity [28, 29, 47, 48]. It has also been indicated that painless heamaturia is among the most common presenting signs of bladder cancer [49, 50]. Approximately 1.3% of patients with asymptomatic microscopic heamaturia (three or more red blood cells per high-power field, in a properly collected specimen, in the absence of an obvious benign cause) will have bladder cancer, with estimates ranging from 0.4% to 6.5% [49]. In a study conducted in Nigeria, Onile et al. [48] observed a close relationship between the intensity of *S. haematobium* infection and the presence of bladder abnormalities.

The identification of *Schistosoma mansoni* eggs in this study might also not be strange, since *S. mansoni* has been described as more invasive, compared to *S. haematobium* [47]. Thus, *S. mansoni* may be found outside where they are frequently observed, which is the superior mesenteric veins in the small intestine [51]. This study did not investigate how coinfection could lead to bladder morbidity as was the case with Lynn et al. [52], where they investigated bladder abnormality in coinfected *Schistosoma* population in Senegal and observed that the presence of *S. mansoni* tends to reduce the risk of *S. haematobium*-associated bladder morbidity. The two cases of mixed infection in this study give a confirmation that *S. mansoni* can be found in urine and that coinfection of *Schistosoma* species may be common in endemic communities.

## 5. Conclusions

Squamous cell metaplasia, inflammatory cells, and hyperkeratosis observed in *S. haematobium*-infected children may play an important role in the disease condition, leading to a severe form of clinical presentation such as bladder cancer in later years, if early attention is not given.

Considering the urinary tract symptoms and the high incidence of bladder carcinoma in *S. haematobium*-endemic areas, it is encouraged that additional diagnostic screening for cancer is appropriate for *S. haematobium*-infected individuals, especially if done at the early stages. Since urine cytology is considered a simple noninvasive procedure used in the diagnosis of urinary tract-associated cancers, its routine use at various healthcare centres on *S. haematobium*-infected patients, especially children, is recommended.

## Figures and Tables

**Table 1:** Observations made in the urine samples after wet preparation microscopy and cytological examinations.

Observations	*n* (%)
Cytological abnormality (*N* = 66)	
Squamous metaplasia	16 (24)
Inflammatory cells	54 (82)
Hyperkeratosis	31 (47)
Egg positivity (*N* = 66)	
*S. h* eggs by wet mount	66 (100)
*S. h* eggs by cytology	40 (61)
Haematuria (*N* = 66)	
RBC (cytology)	22 (33.3)
RBC (R/E-wet prep)	29 (44)
RBC (urine chemistry)	37 (56.0)
Blood intensity by urine dip stick (*N* = 37)	
Trace	8 (12.1)
+	5 (7.5)
++	10 (15.1)
+++	14 (21.2)

## Data Availability

The hard copies and electronic data used to support the findings of this study are available from the corresponding author upon request via patborket2002@yahoo.com or pbtetteh-quarcoo@ug.edu.gh.
